# Facilitators and barriers for performing comprehensive medication reviews and follow-up by multiprofessional teams in older hospitalised patients

**DOI:** 10.1007/s00228-020-02846-8

**Published:** 2020-02-19

**Authors:** Thomas Gerardus Hendrik Kempen, Amanda Kälvemark, Maria Sawires, Derek Stewart, Ulrika Gillespie

**Affiliations:** 1 Hospital Pharmacy Department, Uppsala University Hospital, Uppsala, Sweden; 2 Department of Medical Sciences, Uppsala University, Uppsala, Sweden; 3 Department of Pharmaceutical Biosciences, Uppsala University, Uppsala, Sweden; 4 College of Pharmacy, Qatar University Health, Qatar University, Doha, Qatar

**Keywords:** Multiprofessional collaboration, Medication reviews, Hospital practice, Older patients, Qualitative, Implementation

## Abstract

**Purpose:**

There is a lack of knowledge about factors that influence the performance of comprehensive medication reviews (CMRs) by multiprofessional teams in hospital practice. This study aimed to explore the facilitators and barriers for performing CMRs and post-discharge follow-up in older hospitalised patients from the healthcare professional perspective.

**Methods:**

Physicians and ward-based pharmacists were recruited from an ongoing trial at four hospitals in Sweden. Semi-structured interviews were conducted with 16 physicians and 7 pharmacists. Interview topics were working processes, resources, competences, medication-related problems, intervention effects and collaboration. The interviews were audio-recorded, transcribed verbatim and thematically analysed using the Consolidated Framework for Implementation Research (CFIR). Identified subthemes were categorised as facilitators or barriers and grouped into overarching main themes.

**Results:**

In total, 21 facilitators and 25 barriers were identified across all CFIR domains and grouped in 6 main themes: (a) CMRs and follow-up are needed, but not in all patients; (b) there is a general belief in positive effects; (c) lack of resources is an issue, although the performance of CMRs may save time; (d) pharmacists’ knowledge and skills are valuable, but they need more clinical competence; (e) compatibility with hospital practice is challenging, and roles and responsibilities are unclear and (f) personal contact at the ward is essential for physician-pharmacist collaboration.

**Conclusion:**

Multiple facilitators and barriers for performing CMRs and post-discharge follow-up in older hospitalised patients exist. These factors should be addressed in future initiatives with similar interventions by multiprofessional teams to ensure successful implementation and performance in hospital practice.

**Electronic supplementary material:**

The online version of this article (10.1007/s00228-020-02846-8) contains supplementary material, which is available to authorized users.

## Introduction

Mismanaged prescribing and inappropriate use of medications among older people are a major cause of avoidable harm in healthcare systems across the world [1–3]. Performing comprehensive medication reviews (CMRs), a structured critical examination of a patient’s medications in relation to the patient’s conditions and preferences, aims to optimise treatment benefit and minimise harm [4, 5]. There is evidence that performing such reviews by a multiprofessional team including a clinical pharmacist can improve medication prescribing and increase appropriate use of medications [6–9]. However, less is known about the effects on hard clinical endpoints, justifying the need for high-quality randomised controlled trials (RCTs) [10].

To fulfil this need, the Medication Reviews Bridging Healthcare (MedBridge; www.clinicaltrials.gov: NCT02986425) trial is currently being performed at four hospitals in Sweden [11]. This RCT aims to study the effects of two interventions compared with usual care on older patients’ health outcomes: (1) a CMR by a ward-based pharmacist in collaboration with the physician and patient during hospital stay; and (2) the same as the first intervention, with the addition of a follow-up phone call by the pharmacist 2–7 days and 1–2 months after hospital discharge, and a medication referral to the patient’s general practitioner (GP) upon discharge if necessary.

Clinical trials of interventions comprising multiple components which interact to produce change, like CMRs, are often criticised because of the difficulty to explain the results without examining the underlying processes and the context in which the interventions were performed [12]. Process evaluations alongside such RCTs are therefore highly recommended [13]. Qualitative approaches, such as interviews and focus groups, are often used within process evaluations to provide detailed information about the implementation and performance of the different intervention components [13]. This information may help to understand why the intervention was or was not effective, which can be used to inform policy decisions and support implementation in daily practice.

Process evaluations and other research involving multiprofessional collaboration including ward-based pharmacists often make use of surveys to study the views of the involved healthcare professionals [14–19]. These survey studies report that physicians and nurses generally are satisfied with the collaboration with the pharmacists and that an increase in the quality and safety of the patients’ medication treatment is perceived. The few available studies with a more in-depth qualitative approach conclude that pharmacists can contribute with their pharmaceutical competences and add value to the ward team, but that organisational problems, such as an improper defined role of the pharmacist and limited availability of pharmacist resources, pose challenges to the implementation of multiprofessional interventions [20–24]. One of these studies explored the working relationships of physicians, nurses and ward-based pharmacists in a rural hospital in northern Sweden after the introduction of a clinical pharmacy service including CMRs [24]. It is unclear to what extent these findings are representative for other hospitals in Sweden. A more in-depth understanding is needed to support implementation of CMRs or similar services by multiprofessional ward teams including pharmacists on a wider scale and to ensure long-term sustainability.

We previously explored older patients’ experiences with, and views on, hospital-initiated CMRs and follow-up calls by ward-based pharmacists, as part of a process evaluation of the MedBridge trial [25]. Older patients generally had positive experiences and views. However, some factors, like the unclear role of the pharmacist and problems with receiving and retaining information, may negatively impact effectiveness within the trial. In this second part of the process evaluation, we aimed to explore the facilitators and barriers for performing CMRs and post-discharge follow-up in older hospitalised patients from the healthcare professional perspective.

## Methods

### Study design and methodological approach

A qualitative design with semi-structured interviews was chosen to gain rich accounts of the healthcare professionals’ perspectives. We have taken an interpretive approach as proposed by Snape and Spencer [26]. That is, we accept that the reality exists independently of individual subjective understanding, but that it is only accessible to the researchers via the participants’ interpretations. The Standards for Reporting Qualitative Research (SRQR) [27] were used to design and report the study.

### Study context

**Fig. 1:**
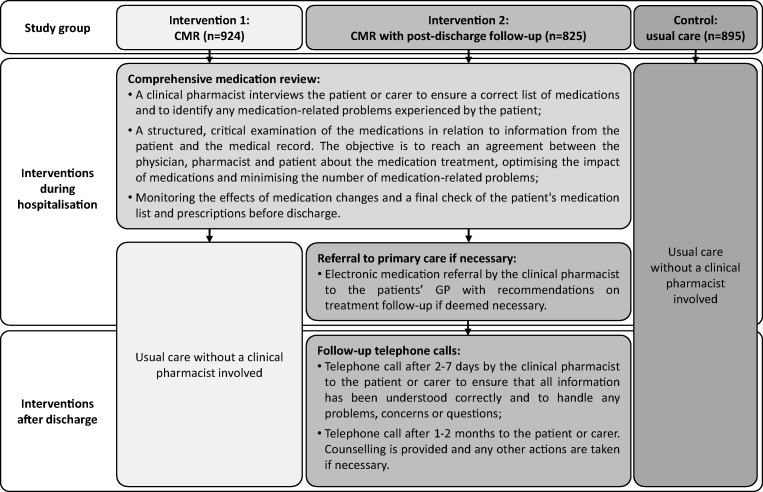
Interventions within the Medication Reviews Bridging Healthcare (MedBridge) trial per study group [11]. CMR, comprehensive medication review; GP, general practitioner

This study was performed in the context of the MedBridge trial, which takes place at in total eight wards within four hospitals in Sweden: the hospitals in Enköping, Gävle, Uppsala and Västerås [11]. The wards differ in terms of medical specialty: geriatric, internal medicine, stroke, diabetes and nephrology. At these wards, the performance of medication reviews by a multiprofessional team including a pharmacist was already established (in Enköping, Gävle and Uppsala) or was implemented about 6 months prior to the start of the trial (in Västerås). The performance of follow-up phone calls and use of medication referrals by pharmacists were introduced and tested approximately 1 to 3 months before the trial started. The pharmacists working at these wards had either completed a full-time 1-year postgraduate programme in clinical pharmacy, or they had completed undergraduate courses in clinical pharmacy and advanced pharmacotherapy. All patients aged 65 years or older who were admitted to one of the wards were asked for informed consent to participate in the MedBridge trial. Patients were excluded if they were in a palliative stage, had been subject to a medication review within the last 30 days, resided in another than the hospital’s region or were admitted for only 1 day. During admission, patients received one of the study interventions or usual care (Fig.1). In total, 2644 patients were included in the MedBridge trial between February 2017 and October 2018. Quantitative results on primary and secondary outcome measures are expected in mid-2020.

### Sampling and recruitment

A purposive sampling approach [28] was used to identify facilitators and barriers that cut across a variety of individuals. In this approach, physicians and pharmacists that had directly been involved in performing the CMRs in the MedBridge trial (intervention 1 or 2) were recruited. We started in Uppsala one month after the start of the trial and aimed to recruit at least eight physicians with different degrees of medical training and positions (at least one consultant, one specialist in training and one junior doctor from both wards) and all four pharmacists. Potential participants were identified by the trial’s local project leader and approached by the researcher who would be conducting the interviews. With those responding positively, a mutually convenient time and place for interview was arranged.

After conducting the interviews and initial analysis in Uppsala, we searched for deviant cases within the other three hospitals: physicians who had expressed scepticism towards collaboration with pharmacists; pharmacists with relatively short and long working experience and pharmacists who had expressed difficulties with performing the interventions throughout the MedBridge trial. An audit trail was kept during recruitment and data analysis.

### Data generation

A discussion guide in Swedish was developed in relation to the intervention components within the MedBridge trial [11] and informed by the concepts of interprofessional collaboration by D’Amour et al. [29]. The topics were working processes, resources, competences, medication-related problems, intervention effects and collaboration (Appendix 1a). Within the interviews, the discussion topics were fixed, whereas the order and exact formulation of the questions were flexible. The interviews in Uppsala were performed by a researcher (MS) who was in the final year of the pharmacy programme and was familiar with the trial’s interventions. The researcher did not have previous professional relationships with the participants. The interviews were conducted in March and April 2017 and lasted between 15 to 40 min. The same researcher who performed the interviews (MS) audio-recorded and transcribed the interviews.

For the additional interviews, the order of the topics in the discussion guide was changed and questions were added and reformulated (Appendix 1b). The interviews in Enköping, Gävle and Västerås were performed by a researcher (AK) with a nursing background, who was working as a research assistant for the MedBridge trial in Uppsala. The researcher was trained in qualitative interviewing and had little to no professional relationship with the participants. Participants were encouraged to be frank and talk freely. Interviews were held between May and October 2018, and lasted between 17 to 104 min (mean: 38 min). The interviews were audio-recorded and transcribed verbatim by one researcher (AK) and checked for transcribing accuracy by another researcher (TK), who was the project coordinator in the MedBridge trial, had a pharmacy background and was trained in qualitative research.

### Data analysis

Thematic analysis was based on the framework approach by Ritchie and Spencer [30] with the Consolidated Framework for Implementation Research (CFIR) [31] to structure the codes. The CFIR is a meta-theoretical framework that has been used in healthcare research to guide evaluation of implementation processes and help to explain outcomes in effectiveness studies [32]. The CFIR consists of five domains, with each domain divided into several constructs: intervention characteristics; outer setting; inner setting; characteristics of individuals; and process (Appendix 2a–c). Two researchers (MS and UG) independently analysed and coded the interview transcripts from Uppsala. One of these researchers (UG) was project leader in the MedBridge trial and had previous experience in qualitative research. The results were then combined into one CFIR-based matrix, and consensus was sought on conflicting results. A third researcher (TK) was available to decide in case no consensus was found. The three researchers (MS, UG and TK) then summarised and interpreted the data within each CFIR construct to create common subthemes in English, which were then categorised as either facilitators or barriers (exemplified in Appendix 2d). Subthemes that could be either a facilitator or a barrier were reformulated and divided into one facilitator and one barrier. For the interviews at the other three hospitals, two researchers (AK and TK) independently coded the interview transcripts followed by the same method as the interviews in Uppsala with a third researcher (UG) involved in identification of subthemes and categorisation into facilitators and barriers. These facilitators and barriers were then matched with those from the interviews in Uppsala, resulting in one integrated framework. As some facilitators and barriers were identified within more than one CFIR construct, these were finally grouped into overarching main themes. Peer scrutiny of the sampling strategy, data generation and analysis was performed by a researcher with extensive experience in qualitative research within healthcare (DS). Research trustworthiness and rigour [33] were addressed in diverse ways throughout the research process (Appendix 3).

## Results

### Demographics of participants

Interviews were held with 16 physicians out of 22 physicians who were invited for participation, and with all 7 pharmacists who were invited. Reasons for not participating were no time for an interview (*n* = 3), too little experience with the interventions (*n* = 1) and no response to the invitation (*n* = 2). All four hospitals were represented among both physicians and pharmacists (Appendix 4). All positions, levels of training and other predefined characteristics were present among the participants, with clinical working experience ranging from a few weeks to over 25 years. Eight physicians were consultants, two were specialists in training and six were junior doctors. Four pharmacists held an additional postgraduate degree in clinical pharmacy.

### Facilitators and barriers

Facilitators and barriers were identified across all five CFIR domains (Appendix 2a and 2b). All findings combined (Appendix 2c) resulted in 21 facilitators and 25 barriers, grouped into 6 main themes (Table 1). Frequent recurring factors and those interpreted as important by the researchers are discussed below and supported by illustrative quotes with the interview number in parentheses (D1–16 for the physicians; P1–7 for the pharmacists).

**Table 1:** Facilitators and barriers for performing comprehensive medication reviews and post-discharge follow-up by ward-based pharmacists in older hospitalised patients grouped into six main themes. These factors were derived from interviews with both physicians and pharmacists, if not stated otherwise

Facilitators	Barriers
CMRs and follow-up are needed, but not in all patients	
• Patients need and appreciate CMRs^I–IV^ • Awareness of legislation and guidelines on CMRs among HCPs^II,IV^ • Need for and willingness to take part in research among HCPs^I,III,V^	• Not all patients want, need or feasible for CMR^I–III,V^ • Pharmacist involvement not always necessary^I^ • Little knowledge about evidence, legislation and guidelines on CMRs among physicians^I,II^
General belief in positive effects of CMRs and follow-up	
• HCPs belief in positive effects of CMRs^I–IV^ • Pharmacist’s work is relevant and appreciated by physicians^I,III,IV^ • CMR more thorough with pharmacist involvement^I,III,IV^ • Positive attitude among pharmacists towards referrals and phone calls^I–III,V^ (only derived from pharmacist interviews)	• Uncertainty among physicians about the long-term effects of CMRs^I^ (only derived from physician interviews) • Insufficient quality of and communication about post-discharge follow-up by primary care^II,III^ • Phone calls may disturb patients^II^ (only derived from pharmacist interviews)
Lack of resources is an issue, although the performance of CMRs may save time	
• CMR or pharmacist may save time and costs^I,III^ • Availability of shared electronic medical record^II,III^	• Lack of time among HCPs^I,III,V^ • No time set for physician-pharmacist contact^I,III,IV^ • CMR takes time for both pharmacist and physician^I,III^ • Phone calls and check upon discharge for all patients is not time efficient^I–V^ (only derived from pharmacist interviews) • Electronic medical record is not complete, fully shared or user-friendly^II,III^
Pharmacists’ knowledge and skills are valuable, but they need more clinical competence	
• Knowledge about the interventions among HCPs^I,IV,V^ • Pharmacist is reliable and has broad pharmaceutical competence^I,III,IV^ • Physicians cannot know everything about medications^IV^ (only derived from physician interviews) • Positive change in physicians’ attitude and knowledge^III,IV^	• Pharmacist lacks or needs more clinical competence^I,III–V^ • Lack of information or training about the interventions and working process among HCPs^III–V^ • Physicians’ competence may decrease^IV^ (only derived from physician interviews)
Compatibility of CMRs with hospital practice is challenging, and roles and responsibilities of ward-based pharmacists are unclear	
• CMR or pharmacist is well-adapted to hospital practice^I,III–V^ • CMR or pharmacist does not interfere with existing work flow^I,III,V^ • Physician has main responsibility^II^ (only derived from physician interviews)	• Hard to fit CMR in hospital practice^I,III,IV^ • Primary care or others responsible and suited for CMR^I,III,IV^ • Pharmacist is not fully integrated in the ward team^III^ • Unclear role of the pharmacist^I–V^ • Pharmacist is dependent on the physician^II–IV^
Personal contact at the ward is essential for physician-pharmacist collaboration	
• Positive experience by physicians with pharmacist collaboration^III,IV^ (only derived from physician interviews) • Presence of pharmacist at the ward and availability^III^ • Personal relationships between HCPs^III^ • Pharmacist participates in medical rounds or meetings^I,III^ • Pharmacist has support from other colleagues^III^ (only derived from pharmacist interviews)	• Pharmacist is not always present at the ward^III^ • Limited contact between pharmacist and consultant physician^III^ • Physicians can feel criticised by the pharmacist^III^ (only derived from physician interviews) • Some physicians less inclined to listen to the pharmacist^III,IV^ • Pharmacist notes in electronic medical record not always appreciated^I,III,V^ • Frequent rotation of HCPs at the ward^I,III^

*CMR* comprehensive medication review, *HCP* healthcare professional

Identified within the CFIR domains [31]:

^I^Intervention characteristics

^II^Outer setting

^III^Inner setting

^IV^Characteristics of individuals

^V^Process

#### CMRs and follow-up are needed, but not in all patients

Physicians and pharmacists believed that the performance of CMRs in older patients with multiple medications is needed. However, not all patients need or appreciate a CMR, and healthcare professionals would prefer to prioritise patients with the highest need.

“You don’t need to go through all patients’ medication lists, but especially the older multimorbid patients with polypharmacy, there I think it’s great and I would like to continue with this [performing CMRs] in the future, if I have to be honest.” (D16)

Awareness of legislation and guidelines imposing the performance of CMRs existed among pharmacists, and to less degree, among physicians. Some physicians stated that a pharmacist may not always be needed as they also conduct CMRs themselves and that other medical specialists can be consulted with medication-related questions.

“For example, if I’m uncertain about antibiotics […] I talk to the infection specialist […] about adjusting dosage and such things.” (D14)

#### General belief in positive effects of CMRs and follow-up

There was a belief that CMRs and follow-up calls could reduce “medication errors” (D11), increase “compliance” (P2) and “patient safety” (D4) and “prevent readmissions” (D3), although the long-term effects on health outcomes were questioned as well. Treatment proposals by pharmacists were “most often relevant” (D15), and the CMRs became “more thorough” (D14) and of “higher quality” (D12) if pharmacists were involved. Although medication referrals and phone calls were considered useful interventions, patients could become “stressed out” (P5) by the phone calls, and there were doubts about the quality of follow-up in primary care, which may reduce the interventions’ effectiveness.

“They [GPs] often accept our referrals, but then they are under much pressure in primary care and a follow-up visit may get forgotten, or it may take a long time until the next visit.” (P1)

#### Lack of resources is an issue, although the performance of CMRs may save time

Lack of time was an important barrier. Finding time for physician-pharmacist contact, monitoring patients during hospital stay and conducting follow-up calls could be “very hard” (P7) and “stressful” (P4) for pharmacists. The performance of CMRs by pharmacists could be time-saving for physicians by supporting them in their work, but it could also increase their workload when medication-related problems were identified during hospital stay.

“Yes, it can take time […] but I think it saves more time than what it takes, so it’s well-invested time.” (D16)

All pharmacists questioned the cost-effectiveness of performing phone calls to all patients, as they felt that many patients did not need or were not feasible for such follow-up.

“I think that the way we’ve worked in the study, calling every patient, is a waste of time. I don’t think that we make best use our competence in that way.” (P5)

Shared electronic record was a major facilitator for communication between physicians and pharmacist and, when available, between hospitals and primary care practices. The pharmacists’ notes could however contain “too much text” (D13), and in-person discussion of treatment proposals was preferred over written communication, because “patients read it all […] and it gets confusing” (D14).

#### Pharmacists’ knowledge and skills are valuable, but they need more clinical competence

Pharmacists were seen as reliable professionals with “broad pharmaceutical competence” (D15), “complementary” (D9) to the physicians’. Their clinical competence could however be improved to provide more clinically relevant input.

“For example, to change antihypertensive medication because of one high blood pressure measurement. Just because the patient is acutely ill today, does not mean that this treatment needs to be changed in the long term.” (D14)

Collaboration with the pharmacist could increase the physicians’ medication-related knowledge, but there was also a risk that physicians would “lose some feeling for it, because they have become accustomed to using pharmacists” (D8). Physicians were generally uninformed about the CMR procedures and about which patients would receive a CMR and post-discharge follow-up. Pharmacists were better informed, but some expressed a lack of training and instructions on how and when to send medication referrals, and how to conduct monitoring and follow-up.

“It feels like we developed some kind of ad hoc method. Right before we started [the trial] it was like: Call the patient and take it from there, just reconcile and make sure the patient has the medications and understood everything.” (P5)

#### Compatibility of CMRs with hospital practice is challenging, and roles and responsibilities of ward-based pharmacists are unclear

Pharmacists had to adapt and fit the CMRs into the existing workflow and often only address medication-related issues that were relevant during hospital stay. It seemed that pharmacists had succeeded doing this, but it was harder at wards where pharmacists had recently been introduced.

“It is important to understand that not everything is relevant to address, and that some things don’t have to be changed right now. You just focus on what’s most relevant to the patient.” (P1)

Some physicians felt that their responsibility was to focus on the cause of admission instead of assessing the patient’s full medication treatment, and that “primary care practices may be better suited” (D9) for performing CMRs. Pharmacists questioned whether they were responsible for the patient’s medication treatment when problems arose during the phone call 1–2 months after discharge. Pharmacists were not fully integrated in the ward team, and their roles, responsibilities and way of working were often not clearly defined. These were major barriers causing “double workload” (P6) and “frustration” (D7). The pharmacists’ dependency on physicians hindered efficiency and “the right to make changes to the medication list” (P7) was proposed to overcome this barrier.

#### Personal contact at the ward is essential for physician-pharmacist collaboration

The physician-pharmacist collaboration was generally positive. Pharmacists were easily accessible. Their presence at medical ward rounds and frequent in-person contact were valued, but this was not common practice at all wards.

“I have been on medical rounds in which the pharmacists did not participate, but came later, and that did not work so well.” (D3)

Building personal relationships was an important facilitator, but frequent staff rotations, pharmacists not always being present at the ward and difficulties reaching the consultant physician, acted as barriers.

“There’s a lot of staff rotation, […] it takes time for them to get integrated in the work method, and to know what they can expect from us and what we can expect of them.” (P4)

Some physicians were more sceptical and less inclined to listen to the pharmacist, but this seemed to improve over time. Junior doctors could sometimes become “a messenger between the pharmacist and consultant physician” (D10), increasing the risk of miscommunication.

## Discussion

This study identified multiple facilitators and barriers, grouped in six main themes, for performing CMRs and post-discharge follow-up by multiprofessional teams in older hospitalised patients. These factors were derived from interviews with physicians and pharmacists, as part of a process evaluation of the MedBridge trial [11].

Physicians and pharmacists believed that older patients need and may benefit from CMRs and post-discharge follow-up. However, due to the design of the MedBridge trial, these interventions were also conducted in patients who might not needed, wanted or were feasible for these interventions, which may have lowered effectiveness and efficiency. Availability of time and resources, including shared electronic medical records, was deemed essential. The CFIR acknowledges the importance of accounting for patient characteristics and needs, prioritisation and the availability of resources [31]. Validated tools to identify patients at high risk for medication-related problems could be used to make more efficient use of available resources [34]. These tools may require self-assessment by the patient [35] or algorithm-based screening of the electronic medical record [36, 37]. Our findings seem consistent with those from qualitative research on CMRs and related interventions by multiprofessional ward teams in other countries and regions [20, 21, 24, 38, 39]. Physicians think that pharmacists are reliable and add knowledge to the team [20, 21, 24]; they perceive positive contribution to the shared goal to improve patient care and safety [21, 38, 39], and presence by pharmacists at the ward is essential to build professional relationships [21, 24]. Theories on interprofessional collaboration and the CFIR emphasise the importance of these facilitators [31, 40]. Although the pharmacists’ knowledge and skills were valued, there was a need for more clinical competence, which is a known area for improvement in pharmacy education worldwide [41, 42]. Pharmacists mentioned being dependent on physicians during the CMR process to make changes to patients’ medication treatment. Physicians also conducted CMRs without pharmacist involvement, but these were less thoroughly performed. Little to no dependency on pharmacists was identified among physicians, whereas interdependency is an important element of successful collaboration [43]. Unlike in the UK, pharmacists in Sweden do not have prescribing rights. Providing these rights and appropriate training to pharmacists may therefore be an opportunity to make the CMR process more efficient and improve collaboration [44].

Flexibility to ensure compatibility with hospital practice was identified as a facilitator in this study. However, it was questioned whether hospital is the most suitable setting to conduct CMRs. Patients may be too ill and not willing to be involved in the CMR process during hospitalisation [25]. Our findings also show that specialist physicians at internal medicine wards may not consider CMRs their responsibility and refer to primary care. GPs may on the other hand not be certain about whose responsibility it is to conduct CMRs either [45].

Multiprofessional collaboration can lead to an improved understanding of the pharmacist’s role in the team [24, 38]. In our study, different perspectives on roles and responsibilities of the pharmacist seemed however to persist. Clarification of tasks and roles is key to successful collaboration and can improve implementation climate [31, 43]. Failure to clarify roles in our trial may be reflected in pharmacists not being perceived as an integrated part of the ward team and physicians not being well-informed about the trial’s interventions and working processes. Consistent with previous research [20, 21, 24, 38, 39], we identified more barriers related to teamwork and collaboration, like pharmacists not always being present at the ward, frequent staff rotations and a lack of time for physician-pharmacist contact. Having pharmacists structurally participate in medical ward rounds may facilitate collaboration. These findings also corroborate our previous research on patient experience with and views on the interventions in the MedBridge trial [25]. Patients valued physician-pharmacist collaboration and the pharmacists’ knowledge and competence, but a lack of understanding about the CMR process and role of the pharmacist may decrease the interventions’ effectiveness.

### Limitations of the study

Despite multiple measures taken to ensure research trustworthiness [33], some limitations to this study exist. Data saturation [46] was not formally assessed, and the interviews in Uppsala were performed during the first and second period (out of six) of the MedBridge trial; hence, relevant data could have been missed. Four out of five researchers had a pharmacy background, which may have led to findings being relatively more positive towards pharmacists. Finally, inherent to qualitative research, the degree of potential influence by the identified facilitators and barriers on the effectiveness of the trial interventions was not measured. We do therefore not know whether the facilitators outweigh the barriers.

### Research and practice implications

This study identified facilitators and barriers which provide possible explanations for why the CMRs and post-discharge follow-up in the MedBridge trial may or may not have been effective. Future initiatives with similar interventions including ward-based collaboration between physicians and pharmacists could address these factors to ensure successful implementation and performance in hospital practice. Our findings emphasise that there is a belief in the need for and benefits of CMRs and post-discharge follow-up in older patients. These interventions should however be adapted to fit hospital practice, perhaps only focusing on medications related to the cause of admission, and tailored to the patients’ individual needs and preferences. Appropriate allocation of time and resources is important, but simply introducing pharmacists to a multiprofessional ward team does not automatically lead to integration. Roles, tasks and responsibilities of the ward team and others involved in patient care in relation to CMRs and post-discharge follow-up should jointly be decided upon.

## Conclusion

Multiple facilitators and barriers for performing CMRs and post-discharge follow-up in older hospitalised patients exist. This study promotes a better understanding of how to address these factors in future initiatives with similar interventions by multiprofessional ward teams, including clinical pharmacists, to ensure successful implementation and performance in hospital practice.

## Electronic supplementary material

ESM 1 (PDF 230 kb)

ESM 2 (PDF 391 kb)

ESM 3 (PDF 227 kb)

ESM 4 (PDF 156 kb)

## Data Availability

The data that support the findings of this study are available on request from the corresponding author. The data are not publicly available due to privacy or ethical restrictions.
