# Supplementary material for: Facilitators and barriers for performing comprehensive medication reviews and follow-up by multiprofessional teams in older hospitalised patients
Source: Eur J Clin Pharmacol. 2020 Feb 19;76(6):775–84. doi: 10.1007/s00228-020-02846-8 (PMC7239809; doi:10.1007/s00228-020-02846-8)
Supplement: Supplementary file 1 — (PDF 230 kb) [file 228_2020_2846_MOESM1_ESM.pdf]

Article title: Facilitators and barriers for performing comprehensive medication reviews and follow-up by multiprofessional teams in older hospitalised patients
Journal name: European Journal of Clinical Pharmacology
Author names: Thomas Kempen, Amanda Kälvemark, Maria Sawires, Derek Stewart and Ulrika Gillespie
E-mail address: thomas.kempen@medsci.uu.se

APPENDIX 1 Discussion guide for the interviews with pharmacists and physicians (translated from Swedish).

Appendix 1a. Discussion guide for the interviews with pharmacists and physicians in Uppsala.

Pharmacists

- To start of, could you tell me about your work background and how long you have worked at this ward?

Working processes
- How do you usually work to bring out the suggestions?
- Could you describe what guidelines you follow in your daily work?
- Do you feel that the physician is accessible if you have the need to discuss something?
- Do you feel that there is a functioning routine in this way of working?

Resources
- Do you usually have enough time to do your job?
- Does it happen that you cannot carry out you work as thorough as you would have wished, and in those cases what are usually the reason for it?
- Wish resources (Electronical medical records etc.) do you usually use to find information you need about patients?
    - How do you think it works?

Competences
- What do you think you contribute with your knowledge?
- What do you think physicians know about medical reviews?
- To what degree do you think physicians should have more knowledge/competence?
- To what degree do you think you should have more knowledge/competence?
    - What kind of competence improvement you like to receive?

Medication-related problems
- If your suggestions in not implemented by the physician, what is usually the reason? How often those this occur?
- How do you reason about what drug related problems to bring up with the physician?
- Do you sometimes disregard drug related problems, and in that case why?
- To what degree do you usually agree with the final results?

Intervention effects
- What impact do You think the medical reviews have on the outcome on the course of the patient's medical care, both on short- and long-term outcomes?
- How would you define the purpose of medical reviews?

- What effect do you think the follow-up calls with patients have?
- What response do you usually get from patients during the calls?
- What do you think the medical referrals to the patient's primary care doctor will lead to?

Collaboration
- What factors do you think is important for a good collaboration with the physician?
- Is there any barriers to the collaboration that we have not talked about?
- Are there any factors that facilitate the collaboration that vi have not talked about?
- Do you have any thoughts on how to improve the collaborations in the future? Or in general, do you have any thoughts on how You could improve the execution of the medical reviews?

Physicians

- To start of, could you tell me about your work background, if you have any specialist training and how long you have worked at this ward?

Working processes
- Could you describe the pharmacist's role and responsibility at the ward?
- Do you know how the pharmacist work to bring out suggestions, and if so, could you describe the process?
- Do you feel that the pharmacist is accessible if you have the need to discuss something?
- Do you feel that there is a functioning routine in this way of working?

Resources
- How often are you in contact with a pharmacist?
- Do you feel there is enough time for meetings with the pharmacist?
    - If no, (wants more time), why do you want more time?
- How frequently do You read the pharmacist notes in the electronical medical record?

Competences
- How familiar are you with medical reviews? Do you know what guidelines there is about medical reviews?
- To what degree do you think pharmacist should have more knowledge/competence?
- To what degree do you think you should have more knowledge/competence?

Medication-related problems
- What types of suggestions does the pharmacist usually come up with?
- If the suggestions in not implemented, what is the suggestions usually about? How often those this occur?
    - What is the most common reason for not making changes?

- Generally, what is your views on the suggestions from the pharmacist?

Intervention effects

- What impact do You think the medical reviews have on the outcome on the course of the patient's medical care, both on short- and long-term outcomes?
- How would you define the purpose of medical reviews?
- Do you have any idea of if there is evidence for medical reviews?

Collaboration

- In which way does the collaboration with the pharmacist affect your work?
- What factors do you think is important for a good collaboration with the Pharmacist?
- Are there any barriers to the collaboration that we have not talked about?
- Are there any factors that facilitate the collaboration that vi have not talked about?
- Do you have any thoughts on how to develop the collaborations in the future?
- Finally, is it anything else you would like to bring up that we haven't already talked about?

Appendix 1b. Discussion guide for the interviews with pharmacists and physicians in Enköping, Gävle and Västerås.

<u>**Pharmacists**</u>

- To start of, could you tell me about your work background and how long you have worked at this ward?

Working processes

- How do you usually work (to bring out the suggestions)?
- Could you describe what guidelines you follow in your daily work?
- Do you feel that there is a functioning routine in this way of working?

Resources

- Do you usually have enough time to do your job?
- Does it happen that you cannot carry out you work as thorough as you would have wished, and in those cases, what are usually the reason for it?
- Which resources (electronical medical records etc.) do you usually use to find information you need about patients?
    - How do you think it works?

Competences

- What do you think you contribute with your knowledge?
- What do you think physicians know about medical reviews?
- To what degree do you think physicians should have more knowledge/competence?
- To what degree do you think you should have more knowledge/competence?

- What kind of competence improvement you like to gain?

Medication-related problems
- If your suggestions in not implemented by the physician, what is usually the reason? How often those this occur?
- How do you reason about what drug related problems to bring up with the physician?
- Do you sometimes disregard drug related problems, and in that case why?
- To what degree do you usually agree with the final results?

Intervention effects
- What impact do You think the medical reviews have on the outcome on the course of the patient's medical care, both on short- and long-term outcomes?
- How would you define the purpose of medical reviews?
- What effect do you think the follow-up calls with patients have?
- What response do you usually get from patients during the calls?
- What do you think the medical referrals to the patient's primary care doctor will lead to?
- What do you think the patients feels about the interventions?
- Is there anything about the patients that could affect the interventions?

Collaboration
- Do you feel that the physician is accessible if you have the need to discuss something?
- What factors do you think is important for a good collaboration with the physician?
- Are there any barriers to the collaboration that we have not talked about?
- Are there any factors that facilitate the collaboration that vi have not talked about?
- Do you have any thoughts on how to improve the collaborations in the future? Or in general, do you have any thoughts on how you could improve the performance of the medical reviews?
- Does the relationship you have with your colleges affect how you work?

Additional questions about the interventions
- How does this differ from other ways of working?
- Do you think there is any better way of working? How?
- Dou you feel that you have received enough information om how to perform these interventions?
- What is your perception of the attitude towards this way of working at your workplace?

Physician

- To start of, could you tell me about your work background, if you have any specialist training and how long you have worked at this ward?

- How long is your experience of working with clinical pharmacists?

Working processes
- What do you know about the Medbridge-trial and its interventions?
- Could you describe the pharmacist's role and responsibility at the ward?
- Do you know how the pharmacist work to bring out suggestions, and if so, could you describe the process?
- Do you feel that the pharmacist is accessible if you have the need to discuss something?
- Do you feel that there is a functioning routine in this way of working?
- Do you work in a different way when the pharmacist is present compared to when they are not (during certain periods)?

Intervention effects
- How familiar are you with medical reviews? Do you know what guidelines there is about medical reviews?
- How would you define the purpose of medical reviews?
- What impact do you think the medical reviews have on the outcome on the course of the patient's medical care, both on short- and long-term outcomes? (Why impact, why not?)
- What (other) reasons could there be for the medical reviews not to have an impact?
- To what degree do you preform medical reviews when the pharmacist is not present? Does it make any difference if the pharmacist in involved?
- What do you think the patients feel about the interventions?

Resources
- When and how often are you in contact with a pharmacist?
- Does the pharmacist save or take your time?
- Do you think the time you have for meetings with the pharmacist is enough?
    - If no (wants more time), why do you want more time?
- How often do you read the pharmacists notes in the electronical medical record?

Competences
- What is your opinion on the pharmacist knowledge/competence?
- What is your opinion on your own knowledge/competence in the same field?

Medication-related problems
- What kind of suggestion does the pharmacist usually have? To what degree are they clinically relevant?
- If the suggestions in not implemented, what is the suggestions usually about? How often those this occur?
    - What is the most common reason for not making changes?
- Generally, what is your views on the suggestions from the pharmacist?

Article title: Facilitators and barriers for performing comprehensive medication reviews and follow-up by multiprofessional teams in older hospitalised patients
Journal name: European Journal of Clinical Pharmacology
Author names: Thomas Kempen, Amanda Kälvemark, Maria Sawires, Derek Stewart and Ulrika Gillespie
E-mail address: thomas.kempen@medsci.uu.se

Collaboration

- In which ways does the collaboration with the pharmacist affect your work?
- What factors do you think is important for a good collaboration with the Pharmacist?
- Are there any barriers to the collaboration that we have not talked about?
- Are there any factors that facilitate the collaboration that vi have not talked about?
- Do you have any thoughts on how improve the collaborations in the future?
- Do you feel that your relationship to the pharmacist affect how you work with them?
- Finally, is it anything else you would like to bring up that we haven’t already talked about?
