# Supplementary material for: Facilitators and barriers for performing comprehensive medication reviews and follow-up by multiprofessional teams in older hospitalised patients
Source: Eur J Clin Pharmacol. 2020 Feb 19;76(6):775–84. doi: 10.1007/s00228-020-02846-8 (PMC7239809; doi:10.1007/s00228-020-02846-8)
Supplement: Supplementary file 2 — (PDF 391 kb) [file 228_2020_2846_MOESM2_ESM.pdf]

Article title: Facilitators and barriers for performing comprehensive medication reviews and follow-up by multiprofessional teams in older hospitalised patients
Journal name: European Journal of Clinical Pharmacology
Author names: Thomas Kempen, Amanda Kälvemark, Maria Sawires, Derek Stewart and Ulrika Gillespie
E-mail address: thomas.kempen@medsci.uu.se

APPENDIX 2 Facilitators and barriers identified within each CFIR domain and underlying construct.

Appendix 2a Facilitators and barriers from the interviews in Uppsala, identified within each Consolidated Framework for Implementation Research (CFIR) domain and underlying construct.[1]

CFIR domain and construct	Facilitators	Barriers
**Intervention characteristics**		
Evidence strength and quality	• Belief in positive outcomes	• Lack of awareness of evidence among physicians
Relative advantage	• Pharmacists' expertise is valued	• Interventions not fully integrated in daily practice
Adaptability	• Flexibility of the healthcare professionals<br>• Interventions adjusted to daily practice	• Lack of time<br>• Different perspective on roles and responsibilities
Complexity	• Participation in medical rounds	• Lack of time<br>• Lack of continuity among physicians
Design quality and packaging	• Interventions adjusted to daily practice<br>• Participation in medical rounds	• Interventions not fully integrated in daily practice
Intervention source; Trialability; Cost	*No subthemes relating to this construct were identified in the interviews*	
**Outer setting**		
Patient needs and resources	• Belief in positive outcomes	• Not all patients benefit
External policies and incentives	• General awareness of focus on elderly	• Lack of awareness of specific policies
Cosmopolitanism; Peer pressure	*No subthemes relating to this construct were identified in the interviews*	
**Inner setting**		
Structural characteristics	• Pharmacists part of the healthcare team<br>• Participation in medical rounds	• Pharmacist's role not always associated with inpatient care
Networks and communications	• Easy communication and continuity<br>• Mutual agreements on therapy	• Interventions not fully integrated in daily practice
Culture	• Positive attitude towards collaboration	• Different perspective on roles and responsibilities
Implementation climate	• Pharmacists part of the healthcare team<br>• Pharmacists' expertise is valued<br>• Mutual goals	• Different perspective on roles and responsibilities<br>• Interventions not fully integrated in daily practice<br>• Lack of feedback<br>• Lack of time
**Characteristics of individuals**		
Knowledge and beliefs about the intervention	• Belief in positive outcomes	• Physicians lack knowledge on how pharmacists exactly work
Self-efficacy	• Flexibility of the healthcare professionals	
Individual stage of change		• Interventions not fully integrated in daily practice
Other personal attributes	• Pharmacists' expertise is valued<br>• Physicians' competence may increase	• Pharmacists' potential is not fully used<br>• Physicians' competence may decrease
Individual identification with organisation	*No subthemes relating to this construct were identified in the interviews*	
**Process**		
Planning		• Need for prioritisation<br>• Lack of time
Engaging		• Different perspective on roles and responsibilities
Executing	• Interventions adjusted to daily practice<br>• Consistency in performance	• Lack of time
Reflecting and evaluation		• Need for evaluation

Article title: Facilitators and barriers for performing comprehensive medication reviews and follow-up by multiprofessional teams in older hospitalised patients
Journal name: European Journal of Clinical Pharmacology
Author names: Thomas Kempen, Amanda Kälvemark, Maria Sawires, Derek Stewart and Ulrika Gillespie
E-mail address: thomas.kempen@medsci.uu.se

Appendix 2b Facilitators and barriers from the interviews in Enköping, Gävle and Västerås, identified within each Consolidated Framework for Implementation Research (CFIR) domain and underlying construct.[1]

CFIR domain and construct	Facilitators	Barriers
**Intervention characteristics**		
Evidence strength and quality	• Need for and willingness to take part in research<br>• Knowledge about the trial and its interventions	• Little knowledge about evidence, legislation and guidelines on CMRs
Relative advantage	• Pharmacist's work is appreciated and relevant<br>• Positive attitude towards referrals and phone calls<br>• CMR more thorough with pharmacist involvement<br>• Pharmacist is reliable and has broad pharmaceutical competence<br>• Belief in positive effects of CMRs<br>• CMR or pharmacist saves time and costs<br>• Patients need and appreciate CMRs	• Phone calls and check upon discharge for all patients is not time efficient<br>• No belief in long-term effects of CMR<br>• Pharmacist involvement not necessary
Adaptability	• Positive attitude towards referrals and phone calls	• Not all patients want, need or feasible for CMR<br>• Pharmacist lacks or needs more clinical competence<br>• Phone calls and check upon discharge for all patients is not time efficient
Complexity		• Primary care or others responsible and suited for CMR<br>• CMR takes time for both pharmacist and physician
Cost	• Belief in positive effects of CMRs<br>• CMR or pharmacist saves time and costs	• CMR takes time for both pharmacist and physician<br>• Phone calls and check upon discharge for all patients is not time efficient
Intervention source; Trialability; Design quality and packaging	*No subthemes relating to this construct were identified in the interviews*	
**Outer setting**		
Patient needs and resources	• Positive attitude towards referrals and phone calls<br>• Belief in positive effects of CMRs<br>• Patients need and appreciate CMRs	• Not all patients want, need or feasible for CMR<br>• Unclear role of the pharmacist<br>• Phone calls and check upon discharge for all patients is not time efficient<br>• Electronic medical record is not complete, fully shared or user-friendly<br>• Phone calls may disturb patients
Cosmopolitanism	• Positive attitude towards referrals and phone calls<br>• Availability of shared electronic medical record	• Electronic medical record is not complete, fully shared or user-friendly<br>• Insufficient quality of and communication about post-discharge follow-up
External policies and incentives	• Physician has main responsibility<br>• Awareness of legislation and guidelines on CMRs	• Pharmacist is dependent on physician<br>• Unclear role of the pharmacist
Peer pressure	*No subthemes relating to this construct were identified in the interviews*	
**Inner setting**		
Structural characteristics		• Frequent staff rotation at the ward<br>• No time set for physician-pharmacist contact

Article title: Facilitators and barriers for performing comprehensive medication reviews and follow-up by multiprofessional teams in older hospitalised patients
Journal name: European Journal of Clinical Pharmacology
Author names: Thomas Kempen, Amanda Kälvemark, Maria Sawires, Derek Stewart and Ulrika Gillespie
E-mail address: thomas.kempen@medsci.uu.se

		• Pharmacist is not always present at the ward<br>• Pharmacist is not fully integrated into the ward team<br>• Limited contact between pharmacist and consultant physician
Networks and communications	• Pharmacist participates in medical rounds or meetings<br>• Pharmacist's work is appreciated and relevant<br>• Pharmacist is reliable and has broad pharmaceutical competence<br>• Pharmacist has support from other colleagues<br>• Availability of shared electronic medical record<br>• Presence at the ward and availability<br>• Positive experience with physician-pharmacist collaboration<br>• Personal relationships	• Lack of time<br>• Pharmacist is dependent on physician<br>• Pharmacist lacks or needs more clinical competence<br>• Frequent staff rotation at the ward<br>• No time set for physician-pharmacist contact<br>• Pharmacist is not always present at the ward<br>• Pharmacist notes in electronic medical record not always appreciated<br>• Limited contact between pharmacist and consultant physician<br>• Pharmacist is not fully integrated in the ward team
Culture	• Pharmacist is reliable and has broad pharmaceutical competence<br>• Positive experience with physician-pharmacist collaboration<br>• Personal relationships	• Pharmacist is dependent on physician<br>• Physicians can feel criticised by the pharmacist<br>• Some physicians less inclined to listen to the pharmacist
Implementation climate	• Pharmacist's work is appreciated and relevant<br>• Positive change in physicians' attitude and knowledge<br>• CMR more thorough with pharmacist involvement<br>• CMR or pharmacist saves time and costs<br>• CMR or pharmacist does not interfere with existing work flow<br>• Belief in positive effects of CMRs<br>• Patients need and appreciate CMRs<br>• Positive attitude towards referrals and phone calls<br>• CMR or pharmacist is well-adapted to hospital practice<br>• Pharmacist is reliable and has broad pharmaceutical competence	• Primary care or others responsible and suited for CMR<br>• Some physicians less inclined to listen to the pharmacist<br>• Pharmacist lacks or needs more clinical competence<br>• Unclear role of the pharmacist<br>• CMR takes time for both pharmacist and physician<br>• Phone calls and check upon discharge for all patients is not time efficient<br>• Hard to fit CMR in hospital practice
Readiness for Implementation	• Need for and willingness to take part in research<br>• CMR more thorough with pharmacist involvement<br>• CMR or pharmacist saves time and costs<br>• Availability of shared electronic medical record	• Lack of time<br>• Pharmacist is dependent on physician<br>• Electronic medical record is not complete, fully shared or user-friendly<br>• Lack of information or training about the trial
**Characteristics of individuals**		
Knowledge and beliefs about the intervention	• Pharmacist's work is appreciated and relevant<br>• Positive change in physicians' attitude and knowledge	• Pharmacist is dependent on physician<br>• Pharmacist lacks or needs more clinical competence<br>• Unclear role of the pharmacist

Article title: Facilitators and barriers for performing comprehensive medication reviews and follow-up by multiprofessional teams in older hospitalised patients
Journal name: European Journal of Clinical Pharmacology
Author names: Thomas Kempen, Amanda Kälvemark, Maria Sawires, Derek Stewart and Ulrika Gillespie
E-mail address: thomas.kempen@medsci.uu.se

	• CMR more thorough with pharmacist involvement<br>• Pharmacist is reliable and has broad pharmaceutical competence<br>• Belief in positive effects of CMRs<br>• Awareness of legislation and guidelines on CMRs<br>• Patients need and appreciate CMRs<br>• Positive experience with physician-pharmacist collaboration<br>• CMR or pharmacist is well-adapted to hospital practice<br>• Knowledge about the trial and its interventions	• Phone calls and check upon discharge for all patients is not time efficient<br>• Lack of information or training about the trial
Self-efficacy	• Physicians cannot know everything about medications<br>• CMR or pharmacist is well-adapted to hospital practice	• Pharmacist lacks or needs more clinical competence
Individual stage of change	• Positive change in physicians' attitude and knowledge	• Some physicians less inclined to listen to the pharmacist<br>• Lack of information or training about the trial
Other personal attributes	• Pharmacist is reliable and has broad pharmaceutical competence<br>• Physicians cannot know everything about medications<br>• CMR or pharmacist is well-adapted to hospital practice	• Primary care or others responsible and suited for CMR<br>• Physicians' competence may decrease
Individual identification with organisation	*No subthemes relating to this construct were identified in the interviews*	
**Process**		
Executing	• Positive attitude towards referrals and phone calls	• Lack of information or training about the trial
Planning; Engaging; Reflecting and evaluation	*No subthemes relating to this construct were identified in the interviews*	

Article title: Facilitators and barriers for performing comprehensive medication reviews and follow-up by multiprofessional teams in older hospitalised patients
Journal name: European Journal of Clinical Pharmacology
Author names: Thomas Kempen, Amanda Kälvemark, Maria Sawires, Derek Stewart and Ulrika Gillespie
E-mail address: thomas.kempen@medsci.uu.se

Appendix 2c Facilitators and barriers from the interviews in Uppsala matched with those from Enköping, Gävle and Västerås.

FACILITATORS	
**Enköping, Gävle and Västerås**	**Uppsala**
**CMRs and follow-up are needed, but not in all patients**	
• Patients need and appreciate CMRs[I-IV]	• General awareness of focus on elderly[II]<br>• Mutual goals[III]
• Awareness of legislation and guidelines on CMRs[II,IV]	• General awareness of focus on elderly[II]
• Need for and willingness to take part in research[I,III]	• Need for evaluation[V]
**General belief in positive effects of CMRs and follow-up**	
• Belief in positive effects of CMRs[I-IV]	• Belief in positive outcomes[I,II,IV]
• Pharmacist's work is appreciated and relevant[I,III,IV]	• Pharmacists' expertise is valued[I,III, IV]
• CMR more thorough with pharmacist involvement[I,III,IV]	
• Positive attitude towards referrals and phone calls[I,II,III,V]	
**Lack of resources is an issue, although the performance of CMRs may save time**	
• CMR or pharmacist saves time and costs[I,III]	
• Availability of shared electronic medical record[II,III]	
**Pharmacists' knowledge and skills are valuable, but they need more clinical competence**	
• Knowledge about the trial and its interventions[I,IV]	• Interventions adjusted to daily practice[I,V]
• Pharmacist is reliable and has broad pharmaceutical competence[I,III,IV]	• Pharmacists' expertise is valued[I,III,IV]
• Physicians cannot know everything about medications[IV]	
• Positive change in physicians' attitude and knowledge[III,IV]	
**Roles, responsibilities and compatibility with clinical practice**	
• CMR or pharmacist is well-adapted to hospital practice[III,IV]	• Flexibility of the healthcare professionals[I,IV]<br>• Consistency in performance[V]<br>• Pharmacists part of the healthcare team[III]
• CMR or pharmacist does not interfere with existing work flow[III]	• Interventions adjusted to daily practice[I,V]
• Physician has main responsibility[II]	
**Healthcare professional communication and collaboration**	
• Positive experience with physician-pharmacist collaboration[III,IV]	• Positive attitude towards collaboration[III]
• Presence at the ward and availability[III]	• Pharmacists part of the healthcare team[III]<br>• Easy communication and continuity [III]<br>• Mutual agreements on therapy[III]
• Personal relationships[III]	• Easy communication and continuity[III]<br>• Positive attitude towards collaboration[III]
• Pharmacist participates in medical rounds or meetings[III]	• Participation in medical rounds[I,III]
• Pharmacist has support from other colleagues[III]	

with corresponding CFIR domains.

Article title: Facilitators and barriers for performing comprehensive medication reviews and follow-up by multiprofessional teams in older hospitalised patients
Journal name: European Journal of Clinical Pharmacology
Author names: Thomas Kempen, Amanda Kälvemark, Maria Sawires, Derek Stewart and Ulrika Gillespie
E-mail address: thomas.kempen@medsci.uu.se

**BARRIERS**	
**Enköping, Gävle and Västerås**	**Uppsala**
**CMRs and follow-up are needed, but not in all patients**	
• Not all patients want, need or feasible for CMR[I,II]	• Need for prioritisation[V]<br>• Lack of time[I,III,V]
• Pharmacist involvement not necessary[I]	
• Little knowledge about evidence, legislation and guidelines on CMRs[I]	• Lack of awareness of evidence among physicians[I]<br>• Lack of awareness of specific policies[II]
**General belief in positive effects of CMRs and follow-up**	
• No belief in long term effects of CMR[I]	
• Insufficient quality of and communication about follow-up after discharge[II]	• Lack of feedback[III]
• Phone calls may disturb patients[II]	
**Lack of resources is an issue, although the performance of CMRs may save time**	
• Lack of time[III]	• Lack of time[I,III,V]
• No time set for physician-pharmacist contact[III]	• Interventions not fully integrated in daily practice[I,III,IV]
• CMR takes time for both pharmacist and physician[I,III]	
• Phone calls and check upon discharge for all patients is not time efficient[I-IV]	• Need for prioritisation[V]
• Electronic medical record is not complete, fully shared or user-friendly[II,III]	
**Pharmacists' knowledge and skills are valuable, but they need more clinical competence**	
• Pharmacist lacks or needs more clinical competence[I,III,IV]	• Different perspective on roles and responsibilities[I,III,V]<br>• Pharmacists' potential is not fully used[IV]
• Lack of information or training about the trial[III-V]	• Physicians lack knowledge on how pharmacists exactly work[IV]<br>• Need for prioritisation[V]
	• Physicians' competence may decrease [IV]
**Compatibility of CMRs with hospital practice is challenging, and roles and responsibilities of ward-based pharmacists are unclear**	
• Hard to fit CMR in hospital practice[III]	• Interventions not fully integrated in daily practice[I,III,IV]
• Primary care or others responsible and suited for CMR[I,III,IV]	
• Pharmacist is not fully integrated in the ward team[III]	• Pharmacists' potential is not fully used[IV]
• Unclear role of the pharmacist[II,III,IV]	• Pharmacist's role not always associated with inpatient care[III]<br>• Physicians lack knowledge on how pharmacists exactly work[IV]<br>• Different perspective on roles and responsibilities[I,III,V]
• Pharmacist is dependent on physician[II-IV]	• Lack of feedback[III]
**Personal contact at the ward is essential for physician-pharmacist collaboration**	
• Pharmacist is not always present at the ward[III]	
• Limited contact between pharmacist and responsible physician[III]	
• Physicians can feel criticised by the pharmacist[III]	
• Some physicians less inclined to listen to the pharmacist [III,IV]	

Article title: Facilitators and barriers for performing comprehensive medication reviews and follow-up by multiprofessional teams in older hospitalised patients
Journal name: European Journal of Clinical Pharmacology
Author names: Thomas Kempen, Amanda Kälvemark, Maria Sawires, Derek Stewart and Ulrika Gillespie
E-mail address: thomas.kempen@medsci.uu.se

• Pharmacist notes in electronic medical record not always appreciated[III]	• Different perspective on roles and responsibilities[I,III,V]
• Frequent staff rotation at the ward[III]	• Lack of continuity among physicians[I]

Appendix 2d Two examples of a quote and the corresponding code, Consolidated Framework for Implementation Research (CFIR) domain and construct, and (sub)thematization.

Quote	Code	CFIR domain – construct	Subtheme – facilitator/barrier	Theme
Physician 9: *"It can sometimes happen that the pharmacist has done a review that is not totally relevant to the cause of admission which could be taken care of in primary care later, and then it just takes time from my working time."*	CMR takes time for physician	Intervention characteristics – cost	CMR takes time for both pharmacist and physician –barrier	Lack of resources is an issue, although the performance of CMRs may save time
Physician 16: *"I also think it makes my job easier because there are moments that I would like to do something but I don't have time, but I know that there is a pharmacist who does it what I think is great, so I think that it saves more than it takes time and that time, I think, is well invested time."*	Pharmacist saves physician's time	Intervention characteristics – cost	CMR or pharmacist saves time and costs – facilitator	Lack of resources is an issue, although the performance of CMRs may save time

Reference

1. Damschroder LJ, Aron DC, Keith RE, Kirsh SR, Alexander JA, Lowery JC. Fostering implementation of health services research findings into practice: a consolidated framework for advancing implementation science. *Implement Sci*. 2009;4:50. doi:10.1186/1748-5908-4-50
