# Supplementary material for: Facilitators and barriers for performing comprehensive medication reviews and follow-up by multiprofessional teams in older hospitalised patients
Source: Eur J Clin Pharmacol. 2020 Feb 19;76(6):775–84. doi: 10.1007/s00228-020-02846-8 (PMC7239809; doi:10.1007/s00228-020-02846-8)
Supplement: Supplementary file 3 — (PDF 227 kb) [file 228_2020_2846_MOESM3_ESM.pdf]

Article title: Facilitators and barriers for performing comprehensive medication reviews and follow-up by multiprofessional teams in older hospitalised patients
Journal name: European Journal of Clinical Pharmacology
Author names: Thomas Kempen, Amanda Kälvemark, Maria Sawires, Derek Stewart and Ulrika Gillespie
E-mail address: thomas.kempen@medsci.uu.se

APPENDIX 3 Facilitators and barriers from the interviews in Uppsala matched with those from Enköping, Gävle and Västerås with corresponding CFIR domains.

**FACILITATORS**	
**Enköping, Gävle and Västerås**	**Uppsala**
**CMRs and follow-up are needed, but not in all patients**	
• Patients need and appreciate CMRs[I-IV]	• General awareness of focus on elderly[II]<br>• Mutual goals[III]
• Awareness of legislation and guidelines on CMRs[II,IV]	• General awareness of focus on elderly[II]
• Need for and willingness to take part in research[I,III]	• Need for evaluation[V]
**General belief in positive effects of CMRs and follow-up**	
• Belief in positive effects of CMRs[I-IV]	• Belief in positive outcomes[I,II,IV]
• Pharmacist's work is appreciated and relevant[I,III,IV]	• Pharmacists' expertise is valued[I,III, IV]
• CMR more thorough with pharmacist involvement[I,III,IV]	
• Positive attitude towards referrals and phone calls[I,II,III,V]	
**Lack of resources is an issue, although the performance of CMRs may save time**	
• CMR or pharmacist saves time and costs[I,III]	
• Availability of shared electronic medical record[II,III]	
**Pharmacists' knowledge and skills are valuable, but they need more clinical competence**	
• Knowledge about the trial and its interventions[I,IV]	• Interventions adjusted to daily practice[I,V]
• Pharmacist is reliable and has broad pharmaceutical competence[I,III,IV]	• Pharmacists' expertise is valued[I,III,IV]
• Physicians cannot know everything about medications[IV]	
• Positive change in physicians' attitude and knowledge[III,IV]	
**Roles, responsibilities and compatibility with clinical practice**	
• CMR or pharmacist is well-adapted to hospital practice[III,IV]	• Flexibility of the healthcare professionals[I,IV]<br>• Consistency in performance[V]<br>• Pharmacists part of the healthcare team[III]
• CMR or pharmacist does not interfere with existing work flow[III]	• Interventions adjusted to daily practice[I,V]
• Physician has main responsibility[II]	
**Healthcare professional communication and collaboration**	
• Positive experience with physician-pharmacist collaboration[III,IV]	• Positive attitude towards collaboration[III]
• Presence at the ward and availability[III]	• Pharmacists part of the healthcare team[III]<br>• Easy communication and continuity [III]<br>• Mutual agreements on therapy[III]
• Personal relationships[III]	• Easy communication and continuity[III]<br>• Positive attitude towards collaboration[III]
• Pharmacist participates in medical rounds or meetings[III]	• Participation in medical rounds[I,III]
• Pharmacist has support from other colleagues[III]	

Article title: Facilitators and barriers for performing comprehensive medication reviews and follow-up by multiprofessional teams in older hospitalised patients
Journal name: European Journal of Clinical Pharmacology
Author names: Thomas Kempen, Amanda Kälvemark, Maria Sawires, Derek Stewart and Ulrika Gillespie
E-mail address: thomas.kempen@medsci.uu.se

**BARRIERS**	
**Enköping, Gävle and Västerås**	**Uppsala**
**CMRs and follow-up are needed, but not in all patients**	
• Not all patients want, need or feasible for CMR[I,II]	• Need for prioritisation[V]<br>• Lack of time[I,III,V]
• Pharmacist involvement not necessary[I]	
• Little knowledge about evidence, legislation and guidelines on CMRs[I]	• Lack of awareness of evidence among physicians[I]<br>• Lack of awareness of specific policies[II]
**General belief in positive effects of CMRs and follow-up**	
• No belief in long term effects of CMR[I]	
• Insufficient quality of and communication about follow-up after discharge[II]	• Lack of feedback[III]
• Phone calls may disturb patients[II]	
**Lack of resources is an issue, although the performance of CMRs may save time**	
• Lack of time[III]	• Lack of time[I,III,V]
• No time set for physician-pharmacist contact[III]	• Interventions not fully integrated in daily practice[I,III,IV]
• CMR takes time for both pharmacist and physician[I,III]	
• Phone calls and check upon discharge for all patients is not time efficient[I-IV]	• Need for prioritisation[V]
• Electronic medical record is not complete, fully shared or user-friendly[II,III]	
**Pharmacists' knowledge and skills are valuable, but they need more clinical competence**	
• Pharmacist lacks or needs more clinical competence[I,III,IV]	• Different perspective on roles and responsibilities[I,III,V]<br>• Pharmacists' potential is not fully used[IV]
• Lack of information or training about the trial[III-V]	• Physicians lack knowledge on how pharmacists exactly work[IV]<br>• Need for prioritisation[V]
	• Physicians' competence may decrease [IV]
**Compatibility of CMRs with hospital practice is challenging, and roles and responsibilities of ward-based pharmacists are unclear**	
• Hard to fit CMR in hospital practice[III]	• Interventions not fully integrated in daily practice[I,III,IV]
• Primary care or others responsible and suited for CMR[I,III,IV]	
• Pharmacist is not fully integrated in the ward team[III]	• Pharmacists' potential is not fully used[IV]
• Unclear role of the pharmacist[II,III,IV]	• Pharmacist's role not always associated with inpatient care[III]<br>• Physicians lack knowledge on how pharmacists exactly work[IV]<br>• Different perspective on roles and responsibilities[I,III,V]
• Pharmacist is dependent on physician[II-IV]	• Lack of feedback[III]
**Personal contact at the ward is essential for physician-pharmacist collaboration**	
• Pharmacist is not always present at the ward[III]	

Article title: Facilitators and barriers for performing comprehensive medication reviews and follow-up by multiprofessional teams in older hospitalised patients
Journal name: European Journal of Clinical Pharmacology
Author names: Thomas Kempen, Amanda Kälvemark, Maria Sawires, Derek Stewart and Ulrika Gillespie
E-mail address: thomas.kempen@medsci.uu.se

• Limited contact between pharmacist and responsible physician[III]	
• Physicians can feel criticised by the pharmacist[III]	
• Some physicians less inclined to listen to the pharmacist [III,IV]	
• Pharmacist notes in electronic medical record not always appreciated[III]	• Different perspective on roles and responsibilities[I,III,V]
• Frequent staff rotation at the ward[III]	• Lack of continuity among physicians[I]
