# Supplementary material for: Facilitators and barriers for performing comprehensive medication reviews and follow-up by multiprofessional teams in older hospitalised patients
Source: Eur J Clin Pharmacol. 2020 Feb 19;76(6):775–84. doi: 10.1007/s00228-020-02846-8 (PMC7239809; doi:10.1007/s00228-020-02846-8)
Supplement: Supplementary file 4 — (PDF 156 kb) [file 228_2020_2846_MOESM4_ESM.pdf]

Article title: Facilitators and barriers for performing comprehensive medication reviews and follow-up by multiprofessional teams in older hospitalised patients
Journal name: European Journal of Clinical Pharmacology
Author names: Thomas Kempen, Amanda Kälvemark, Maria Sawires, Derek Stewart and Ulrika Gillespie
E-mail address: thomas.kempen@medsci.uu.se

APPENDIX 4 Number of participating physicians and pharmacists acting as participants per level of clinical training and hospital, and length of clinical working experience per profession.

Training, number	Hospital, number	Working experience, range
**Physicians, 16**		
• Consultant specialist, 8<br>• Specialist in training, 2<br>• Junior doctor, 6	• Enköping, 2<br>• Gävle, 3<br>• Uppsala, 8<br>• Västerås, 3	2 weeks – 25 years
**Pharmacists, 7**		
• Post-graduate clinical pharmacy programme, 4<br>• No formal post-graduate clinical programme, 3	• Enköping, 1<br>• Gävle, 1<br>• Uppsala, 4<br>• Västerås, 1	2 years – 9 years
